# Applications and prospects of artificial intelligence in the auxiliary diagnosis of pediatric pulmonary tuberculosis

**DOI:** 10.3389/fmicb.2025.1738926

**Published:** 2026-01-13

**Authors:** Xingyu Lu, Yiyi Hu, Yue Hu, Fei Zhao, Peiyang Fan, Yingyu Luo, Juan Li

**Affiliations:** 1 Department of Healthcare-Associated Infection Management, West China Second University Hospital of Sichuan University/West China Women's and Children's Hospital, Chengdu, China; 2 Key Laboratory of Birth Defects and Related Diseases of Women and Children, Sichuan University, Ministry of Education, Chengdu, China

**Keywords:** artificial intelligence, auxiliary diagnosis, multimodal fusion, pediatric pulmonary tuberculosis, tuberculosis

## Abstract

Tuberculosis (TB) is a serious disease that poses a significant threat to the health of children and adolescents, with *pulmonary tuberculosis* (PTB) being the most common type. Due to the lack of specificity in clinical manifestations and symptoms, early screening and diagnosis of pediatric pulmonary tuberculosis present significant challenges. In recent years, the *artificial intelligence* (AI) healthcare industry has emerged as a major driving force for transformation in the global healthcare sector. Through technologies such as deep learning, natural language processing, computer vision and multimodal fusion, intelligent solutions are brought to medical links such as clinical auxiliary diagnosis. The combination mode of AI with medical imaging, laboratory diagnosis, pathology examination and other data has also been gradually applied to tuberculosis screening and diagnosis. However, its development is constrained by bottlenecks such as the scarcity of high-quality data on children, insufficient interpretability of models, lack of external validation, and unclear clinical translation paths. Moreover, most of the existing related studies focus on adult pulmonary tuberculosis, and there is a lack of sufficient research and reporting on pediatric pulmonary tuberculosis. This article aims to systematically review the research and application status of AI in the auxiliary diagnosis of pediatric pulmonary tuberculosis in recent years, critically analyze the current limitations, and explore that in the future, efforts should be made to build cross-institutional and multi-center collaborative datasets and carry out explainable AI verification centered on clinical efficacy. Explore the development path of the application of AI in the full-chain management of “prevention—diagnosis—treatment—management” of pediatric pulmonary tuberculosis.

## 1 Introduction

Tuberculosis is a chronic respiratory infectious disease caused by *Mycobacterium tuberculosis* infection and has long been a major challenge in the global public health field (Lewinsohn et al., 2017). According to the Global tuberculosis report 2025 by the WHO, global tuberculosis prevention and control has made positive progress despite continuous challenges, and most indicators are on the right track. Despite this, tuberculosis remains a major public health problem worldwide, one of the top ten causes of death globally, and the leading cause of death from a single source of infection (World Health Organization, 2025). It is estimated that there will be approximately 10.7 million new tuberculosis patients worldwide in 2024, a decrease of 2.2% compared to 2023. Among them, 1.177 million patients are children (aged 0–14), accounting for 11% of the total cases. In terms of regional distribution, the new tuberculosis patients in 2024 are mainly distributed in the Southeast Asia region (34%), the Western Pacific region (27%), the African region (25%), the Eastern Mediterranean region (8.6%), the American region (3.3%), and the European region (1.9%) as defined by the World Health Organization. It is estimated that there will be 1.23 million deaths from tuberculosis worldwide in 2024, among which 15.9% are children and adolescents. It is speculated that improper diagnosis and treatment are the main causes of death for most children with tuberculosis (World Health Organization, 2025; Dodd et al., 2017).

In addition, the tuberculosis epidemic varies greatly among different countries around the world, which is related to the regional differences in disease burden. The majority of tuberculosis cases with an estimated incidence rate of less than 10 per 100,000 are distributed in the American and European regions, while the rest are distributed in the Eastern Mediterranean and Western Pacific regions. Most of the 30 countries with a high burden of tuberculosis are located in Southeast Asia, with estimated incidence rates ranging from 150 per 100,000 to 400 per 100,000. Among them, the estimated incidence rates in countries such as Lesotho, Papua New Guinea and the Philippines exceed 500 per 100,000. It is worth noting that the number of tuberculosis cases among children varies greatly among countries and regions where tuberculosis is prevalent. This is caused by the combined effect of multiple social factors, including socioeconomic factors, population factors, environmental factors, medical resource factors, tuberculosis prevention and control strategies and policy factors, etc. These factors can lead to shortages of medical resources, poor sanitary conditions, insufficient professional level of medical personnel, unreasonable treatment plans and inadequate tuberculosis prevention and control planning in high-burden areas, thus making it impossible to detect and treat children with tuberculosis in a timely manner, and increasing the possibility of underreporting and misdiagnosis. Tuberculosis cases reported among children (aged 0–14) account for less than 2% of all reported tuberculosis cases in Vietnam, while in Papua New Guinea it is 25% (World Health Organization, 2025; Dodd et al., 2017; Li et al., 2024; Hu and Gao, 2024). Therefore, pediatric tuberculosis remains an important issue related to the public health of children and adolescents.

**Figure 1:**
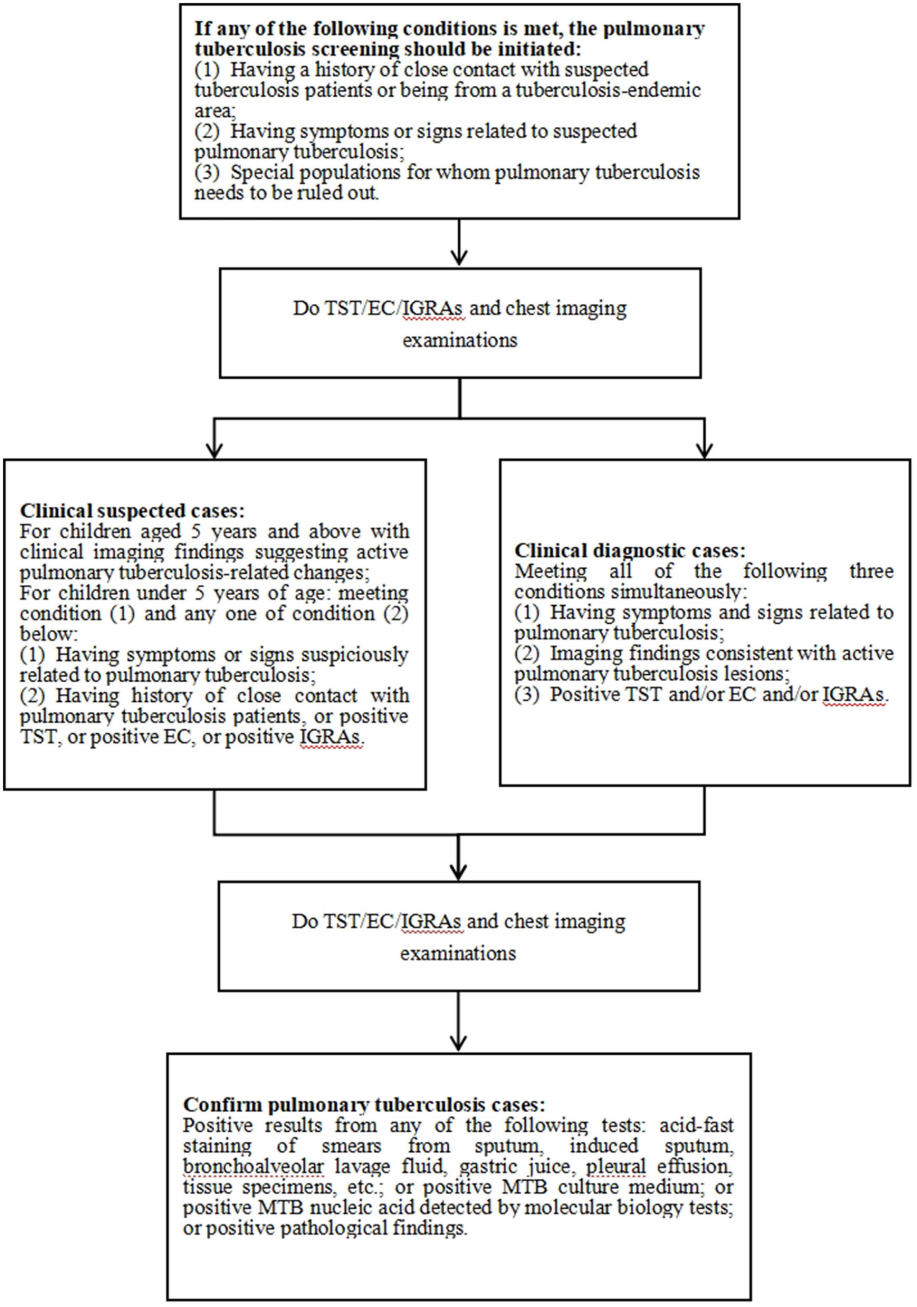
The flowchart for pediatric pulmonary tuberculosis diagnosis. TST, tuberculin skin test; EC, recombinant *Mycobacterium tuberculosis* fusion protein; IGRAs, interferon-gamma release assay; MTB, *Mycobacterium tuberculosis*.

Pediatric tuberculosis is most common in children under 5 years old, with 70%−80% being pulmonary tuberculosis. Compared with older children, children under 5 years old have a higher risk of undiagnosed tuberculosis and developing serious diseases (Marais et al., 2005; Liang et al., 2025). Compared with adults, the clinical symptoms of pediatric pulmonary tuberculosis are complicated and lack specificity. The positive rate of pathogen detection is low and it takes a considerable amount of time. However, molecular biology methods are expensive and have low popularity, which makes early screening and clinical diagnosis still full of challenges (Howard-Jones and Marais, 2020). In March 2022, the WHO consolidated guidelines on tuberculosis (Module 5): Management of tuberculosis in children and adolescents highlighted the update of Xpert MTB/RIF Ultra as an initial diagnostic and rifampicin resistance detection tool, and adopted an integrated treatment decision-making pathway as the initial diagnostic method for children with pulmonary tuberculosis signs and symptoms (World Health Organization, 2022). In April 2022, the Chinese Journal of Applied Clinical Pediatrics released the Expert Consensus on the diagnosis of pediatric pulmonary tuberculosis, presenting the diagnostic process for pediatric pulmonary tuberculosis (Figure 1), emphasizing the importance of basic laboratory examinations and imaging examinations, and the cautious use of invasive examinations (Wan and Shen, 2022). These play an important role in the early diagnosis of pediatric pulmonary tuberculosis.

In recent years, breakthroughs in artificial intelligence (AI) in medical image analysis and multimodal data fusion have opened up new opportunities for improving the diagnostic efficiency of pediatric pulmonary tuberculosis. AI can process and analyze big data, accelerate the disease diagnosis process, enhance work efficiency, and simultaneously reduce the risks of human misdiagnosis and missed diagnoses. In the field of tuberculosis, most AI-related research has primarily focused on adult tuberculosis, with limited reports on pediatric pulmonary tuberculosis (Luo et al., 2023). This review objectively summarizes the technical application and existing challenges of artificial intelligence in the auxiliary diagnosis of pediatric pulmonary tuberculosis, and explores its future development path in actual clinical practice.

## 2 Methods

### 2.1 Nature and scope

This study is a narrative review, aiming to systematic arrangement, summarize and critically discuss the research progress, technical paths and future challenges of AI in the field of auxiliary diagnosis of pediatric pulmonary tuberculosis. Unlike systematic reviews, this study does not conduct quantitative meta-analysis but focuses on providing a comprehensive qualitative elaboration of the existing evidence.

### 2.2 Retrieval methods

To ensure coverage of the core literature in this field, we adopted a multi-source search strategy. We searched PubMed, Web of Science, Scopus, and CNKI databases, mainly focusing on the literature published from 2010 to 2025 to reflect the latest research progress in this field. At the same time, foundational key literature in this field is not subject to this time limit. The core search terms include tuberculosis, pulmonary tuberculosis, pediatric tuberculosis and pediatric pulmonary tuberculosis related to the disease; children, infants, pediatrics and adolescents related to the population; artificial intelligence, AI, machine learning, deep learning, computer vision, natural language processing, auxiliary diagnosis, and diagnosis related to technology.

### 2.3 Screening and inclusion

We mainly included original research, important reviews and authoritative guidelines exploring the application of AI technology in the auxiliary diagnosis of pediatric pulmonary tuberculosis. Exclude literature that is irrelevant to the topic, merely an abstract of the conference, or for which the full text cannot be obtained. The search results were imported into the file management software to remove duplicate records. The first author conducted a preliminary screening of the titles and abstracts to exclude studies that clearly did not meet the inclusion criteria. Conduct a full-text intensive reading of the potential included literature. Through discussion, determine the core literature set to be ultimately included in the review and have it verified by the corresponding author. This process aims to ensure that the included studies can represent the main technical paths and application scenarios of AI in the auxiliary diagnosis of pediatric pulmonary tuberculosis.

### 2.4 Integration

We integrated the final included literature using the thematic analysis method. Based on the types of AI technologies and their specific application scenarios in the diagnosis of pediatric pulmonary tuberculosis, the research findings are summarized, compared and critically discussed, aiming to sort out a clear technological development trajectory, identify current research consensus and gaps, and ultimately form a narrative logic pointing to future challenges and research directions.

## 3 The diagnostic dilemma in pediatric pulmonary tuberculosis

### 3.1 The complexity and latency of the disease's characteristics

Primary pulmonary tuberculosis is the main form of pediatric pulmonary tuberculosis. Compared to adults, pediatric pulmonary tuberculosis often presents with an insidious onset and diverse symptoms, lacking typical clinical manifestations such as coughing or fever, especially in the early stages of the disease when symptoms may be absent or difficult to recognize. Some children may exhibit recurrent respiratory infections, unexplained weight loss, or slow weight gain, and it is difficult to make a diagnosis based solely on clinical manifestations. Extra pulmonary tuberculosis is more common in children and is prone to hematogenous dissemination, affecting various organs throughout the body, such as the brain, bones and joints, abdominal cavity, and pericardium. This results in diverse and non-specific clinical manifestations, increasing the difficulty of diagnosis. The clinical manifestations of pediatric pulmonary tuberculosis are also easily masked by other underlying diseases, such as pneumonia, human immunodeficiency virus infection, and malnutrition, which can easily lead to misdiagnosis (Wu et al., 2012; Tao et al., 2019; Zhang et al., 2014; Jaganath et al., 2022; Chiappini et al., 2016). World Health Organization (WHO) noted in its 2023 edition of “The Roadmap toward ending TB among children and adolescents, third edition” that the misdiagnosis and missed diagnosis rates for pediatric pulmonary tuberculosis under 15 years of age reach 45%, and as high as 58% in children under 5 years of age (World Health Organization, 2023).

### 3.2 Significant difficulties in obtaining etiological evidence

#### 3.2.1 Difficulty in specimen collection

Sputum, gastric fluid, bronchoalveolar lavage fluid, cerebrospinal fluid, and other specimens are ideal specimens for diagnosing pediatric pulmonary tuberculosis, tracheobronchial tuberculosis (TBTB), and tuberculous meningitis (TBM). However, collecting these specimens from children presents certain challenges. Children produce small amounts of sputum and have weak coughing strength, especially infants and toddlers, who often cannot expel sputum, making sputum specimen collection difficult (Lamb and Starke, 2016; World Health Organization, 2021a; Basile et al., 2022). A gastric fluid specimen is a better option for improving the detection rate of pathogens, as children who cannot expectorate often swallow sputum into the stomach. However, the collection of gastric fluid is an invasive procedure that requires high cooperation from parents and professional training for medical staff, thereby increasing the difficulty of obtaining pathogen evidence (Kizito et al., 2018). From the perspective of AI model development, this clinical reality directly leads to a severe shortage of high-quality training data, making it difficult to obtain large-sample training datasets. However, the use of limited small sample datasets restricts the performance and generalization ability of AI models, making it difficult to cover the true distribution and diversity of the data, and resulting in the models being unable to fully learn universal laws. Secondly, the risk of overfitting of the model increases. It over adaptation to the noise and details in small sample data while ignoring the essential features of the data, resulting in a significant decline in performance in test data or practical applications. Small sample datasets may also lead to results such as insufficient feature learning of the model, decreased model stability, increased prediction uncertainty, and unreliable evaluation.

#### 3.2.2 Existing diagnostic techniques have certain limitations

Another difficulty in the diagnosis of pediatric tuberculosis is the low bacterial load in specimens, so the positive rate of etiological detection is much lower than that in adults. Due to insufficient specimen collection, the positive rates of acid-fast staining, culture and nucleic acid molecular diagnosis of specimens are also relatively low (Lamb and Starke, 2016; Nicol et al., 2015; Nicol and Zar, 2020). The sensitivity of smear microscopic examination in pediatric tuberculosis is less than 15% (Wu et al., 2012). MTB culture is the gold standard for diagnosing TB, but its sensitivity is only 20.0%−46.4% (Wu et al., 2012; Tao et al., 2019; Zhang et al., 2014). Although the WHO recommends Xpert MTB/RIF for the early diagnosis of pediatric tuberculosis and can replace smears, cultures, etc., for the rapid diagnosis of extrapulmonary TB, its sensitivity in the diagnosis of pediatric pulmonary tuberculosis is only 51%−66% (Yin et al., 2014; Detjen et al., 2015). In addition, this method requires expensive instruments, reagents, consumables; its promotion and application in primary hospitals are also subject to certain restrictions. The sensitivity of the new-generation Xpert MTB/RIF Ultra is significantly better than that of Xpert MTB/RIF. However, the relevant research data in children with different types of extrapulmonary TB and children with HIV infection are still scarce, and it has not yet been widely applied globally (Sun et al., 2019; Kay et al., 2022; Sun et al., 2020).

A multicenter study showed that the sensitivity of the sputum Ultra test in children diagnosed with pulmonary tuberculosis through culture was 64.3% (Sabi et al., 2018). Nicol et al. (2018) showed that the Ultra-sensitivity was 75.3% in children with positive sputum MTB culture. Secondly, immunology-related tests, TST and IGRAs, have certain value in auxiliary diagnosis of pediatric tuberculosis, but false negatives can also occur in children with weakened or suppressed immune function and severe tuberculosis (Chin et al., 2023). Therefore, future research should further explore more convenient, rapid, economical, and practical laboratory testing methods suitable for children to improve the accuracy and practicality of diagnosing pediatric tuberculosis.

### 3.3 Clinicians in primary hospitals do not have sufficient clinical experience and attention to pediatric pulmonary tuberculosis

Pediatric pulmonary tuberculosis can manifest in various forms, including primary, miliary, invasive, and endobronchial tuberculosis. Especially in young children, symptoms may be atypical, and early clinical manifestations can easily be confused with respiratory infections (bacterial pneumonia, mycoplasmal pneumonia, viral pneumonia), asthmatic diseases (capillary bronchitis, asthma, etc.), langerhans cell histiocytosis, allergic reaction alveolitis, and other interstitial lung diseases, or even malignant tumors (Wu et al., 2012; Miranda-Schaeubinger et al., 2023). Additionally, the general population in middle- and low-income countries still lacks sufficient understanding of the signs and symptoms of tuberculosis, preventive measures, and treatment means. Clinicians in primary hospitals have low awareness of tuberculosis-related knowledge, often neglecting to differentiate tuberculosis in clinical diagnosis and differential diagnosis, resulting in insufficient clinical diagnostic capabilities (Craciun et al., 2023). Survey studies have found that only 43.7% of pediatric medical workers have received tuberculosis knowledge training, and awareness of tuberculosis prevention strategies is generally low. Since these medical institutions are often the first point of contact for pediatric tuberculosis patients, this increases the likelihood of misdiagnosis or missed diagnosis of pediatric pulmonary tuberculosis (Yu et al., 2020; Chen et al., 2022). Moreover, the direct impact of this clinical reality on AI models is that label noise caused by diagnostic errors, uncertainties or incompleteness may lead to problems such as weakened classification accuracy and generalization ability of AI models, unstable training processes, reduced model robustness, distorted evaluation metrics, and decreased data utilization. Effective measures need to be taken to deal with label noise in order to improve the performance and reliability of the model. In addition, during the growth and development of children, their bone structure, organ morphology and physiological functions are constantly changing, which leads to significant age-related variability in radiological images. This variability requires that AI models have stronger generalization capabilities to adapt to the imaging features of different age groups and growth stages. The physiological characteristics and disease spectra of children are different from those of adults, and the annotation of children's radiological images may be more complex, increasing the difficulty of annotation and the complexity of data quality control.

In addition, there are many rare diseases among children. This makes it somewhat difficult to obtain sufficient training data, which may lead to poor performance of the model in diagnosing rare diseases and result in false positive or false negative outcomes. Due to the fact that different hospitals use different imaging equipment, there may be differences in equipment performance, scanning parameters and imaging algorithms, resulting in different image quality and technical characteristics. AI models need to adapt to the image differences caused by different devices and imaging parameters. Otherwise, it will affect the unstable performance of the model on the images collected by different devices. The diagnosis and treatment of pediatric pulmonary tuberculosis require a comprehensive consideration of multiple factors such as growth and development, genetic factors, and environmental factors. When providing diagnostic suggestions, AI models need to be combined with the judgments of clinical doctors and deeply integrated with the clinical workflow to fully leverage their auxiliary role.

### 3.4 The imbalance of medical resources and the persistence of delayed diagnosis

Tuberculosis prevention and control fall under public health medical services. Research indicates that there are gaps and deficiencies in the general population of middle- and low-income countries regarding knowledge, attitudes, and behaviors related to tuberculosis. Additionally, barriers to accessing healthcare services and the stigmatization and isolation associated with tuberculosis are reasons why people do not frequently utilize healthcare services when symptoms arise, leading to delayed diagnosis of tuberculosis (Craciun et al., 2023). Some developing countries and impoverished regions may face shortages of tuberculosis diagnostic resources (such as a lack of radiologists), while some developed countries place greater emphasis on early diagnosis and treatment of tuberculosis.

Taking China as an example, the current level of tuberculosis prevention and control in China is better in the east than in the west, and better in urban areas than in rural areas. Tuberculosis clinics responsible for the diagnosis and treatment of tuberculosis across the country generally face issues such as inadequate infrastructure, outdated laboratory equipment, and low adoption rates of new diagnostic technologies, resulting in relatively weak grassroots prevention and control capabilities (Xu et al., 2020; Long et al., 2021). Additionally, the fifth national tuberculosis epidemiological survey in 2010 showed that over 50% of tuberculosis patients in China experienced delayed diagnosis (Wang et al., 2012). Two studies in 2017 indicated that the median delay in seeking medical attention for tuberculosis patients in China was 44 days, and the median delay in diagnosis was 20 days (Wang et al., 2017; Chimbatata et al., 2017). The causes of delayed diagnosis in tuberculosis patients include the first visit to medical institutions with inadequate infrastructure and lacking sputum culture capabilities, living too far from medical institutions, and a lack of proper understanding of tuberculosis. Therefore, improving the diagnostic and treatment conditions and capabilities of primary medical institutions, enhancing patients' health awareness, and reducing delayed diagnosis are major challenges in tuberculosis prevention and control.

## 4 The applications of AI in the auxiliary diagnosis of tuberculosis

### 4.1 Medical imaging

Early screening and diagnosis of pulmonary tuberculosis are crucial for the control of the disease. Nowadays, computer-aided detection and diagnosis based on AI algorithms have become an active area of research in medical imaging. The WHO recommends the use of chest X-rays as a screening technique (World Health Organization, 2021b). The AI products developed in the early stage for assisting in the diagnosis of pulmonary tuberculosis were computer-aided diagnosis (CAD) systems established using manually created preset feature models, which were used to achieve automatic recognition and diagnosis of lesions (Jaeger et al., 2013; Nijiati et al., 2021; Lure et al., 2017).

Palmer et al. (2023) used CAD4TB software with a fine-tuning program to evaluate the program's performance based on chest X-ray images of 620 children under the age of 13 with pulmonary tuberculosis. They found that the AUC increased from 0.58 to 0.72 after incorporating the fine-tuning program. They suggested that CAD could serve as an alternative to manual interpretation of chest X-rays and potentially provide an additional diagnostic tool for pediatric tuberculosis, thereby enabling early clinical diagnosis and timely treatment, which could improve outcomes for pediatric tuberculosis patients and reduce mortality rates. However, the performance of CAD systems is easily influenced by artificial predefined features, such as the presence of cavities, making it challenging to incorporate various manifestations of tuberculosis into a single CAD system. Additionally, the datasets used by CAD systems are relatively small, resulting in overall low performance, which limits their applicability in different sanitary conditions worldwide (Jaeger et al., 2013; Kulkarni and Jha, 2020).

To address issues such as small datasets and low clinical efficacy in traditional CAD systems, deep learning—a branch of machine learning has increasingly demonstrated its exceptional potential in medical image analysis. For example, convolutional neural networks (CNNs) have shown particular promise in analyzing various lung lesions in the medical field, especially in the diagnosis of tuberculosis (Santosh et al., 2022). CNNs are artificial intelligence models specifically designed for image analysis in deep learning. They convert pixel-level numerical information into an understanding of image content through a series of input layer, convolutional layer, pooling layer, fully connected layer, and output layer. They mimic the functions of human brain neurons by iteratively training on image data, automatically selecting relevant features, and optimizing parameters until the optimal model is obtained (Ngiam and Khor, 2019). In medical image analysis, CNNs learn by “viewing” thousands of annotated images, automatically identifying visual features that distinguish different diagnostic outcomes during this process (Hong et al., 2017). Additionally, numerous commercial deep learning AI software tools for diagnosing pulmonary tuberculosis have been developed both domestically and internationally, such as qXR (India), Lunit INSIGHT CXR (South Korea), Aidoc (Israel), and JF CXR-1 (China). Currently, five AI algorithms have obtained certification for tuberculosis detection in chest X-rays. However, only qXR and CAD4TB achieve specificity of at least 70% at a sensitivity of 90% (specificity were 74.3% and 72.9%, respectively), meeting the WHO's target product profile (TPP) criteria for molecular diagnostic tests (Qin et al., 2021).

Hwang et al. (2016) used a data set of 10,848 chest X-ray images from the Korean Tuberculosis Research Institute. Seventy percentage of the data set was used to train a deep convolutional neural network model called AlexNet, 15% was used for diagnosing pulmonary tuberculosis, and the results were compared with those from 138 chest X-ray images from the National Institutes of Health (NIH) and 662 chest X-ray images from Shenzhen Third Hospital. The area under the AUC for AlexNet in diagnosing pulmonary tuberculosis reached 0.96 on the KIT data set, 0.88 on the NIH data set, and 0.93 on the Shenzhen Third Hospital data set. Additionally, a study involving 846 patients (Ma et al., 2020) used the U-Net deep learning model based on CNN as the basic framework to develop an automated tuberculosis detection model based on CT images for screening active tuberculosis, achieving an AUC value of 0.98. Another study applied deep convolutional neural networks (DCNN) to chest X-rays to distinguish between active pulmonary tuberculosis and non-tuberculosis patients, including 5,000 tuberculosis patients and 4,628 non-tuberculosis patients. Three different DCNN algorithms—AlexNet, VGG, and ResNet—were trained, and they obtained AUC values of 0.9917, 0.9902, and 0.9944, respectively. Among these, the AI based on the ResNet algorithm demonstrated outstanding diagnostic capabilities across different clinical subgroups and accurately marked tuberculosis regions on chest X-rays, outperforming other models (Nijiati et al., 2022). These results indicate that deep learning models perform well in the diagnosis of screening for active pulmonary tuberculosis.

In addition to efforts in tuberculosis screening and diagnosis, AI is particularly important in distinguishing tuberculosis from other pulmonary diseases. Huang et al. (2020) developed a novel diagnostic method for tuberculosis by integrating a deep transfer CNN and extreme learning machine (ELM), achieving accuracy, sensitivity, and specificity of 94.57%, 93.69%, and 95.15%, respectively, for lung nodule diagnosis. Hu and Bian (2023) developed a predictive model for F-FDG PET/CT based on radioactive features and validated its predictive value in distinguishing solitary lung adenocarcinoma from tuberculosis. Wang et al. (2021) collected 301 cases and established an unlabeled 3D-ResNet model to distinguish non-tuberculous mycobacterial disease from tuberculosis. The model achieved accuracy rates of 90%, 88%, and 86% on the training, validation, and test sets, respectively (sensitivity = 0.92). The ML model XmarTB trained by Parreira et al. (2024) Can accurately diagnose automatically detected abnormal CXR and can well distinguish pulmonary tuberculosis from other lung diseases. This tool holds significant promise in helping to proactively detect tuberculosis cases and providing rapid and accurate support for early diagnosis. In addition, there are research reports on the use of artificial intelligence to distinguish between pulmonary tuberculosis and community-acquired pneumonia, pulmonary tuberculosis and lung cancer, and drug-resistant pulmonary tuberculosis, all of which have yielded promising results.

As can be seen, deep learning holds great promise for the diagnosis of pulmonary tuberculosis, both on chest X-rays and CT scans. The World Health Organization's Tuberculosis Operational Manual has also added a new recommendation for the use of computer-aided diagnosis to replace manual interpretation of chest X-rays for the screening of pulmonary tuberculosis in individuals aged 15 years and older (World Health Organization, 2021b). However, there is an urgent need for standardized reporting guidelines for pulmonary tuberculosis AI clinical trials to further confirm their stability and heterogeneity across various populations and environments (Li and Lü, 2024).

In addition, with the rise of visual converters (Vits) and their derivative models, researchers have also begun to apply them to the image analysis of age-related macular degeneration (AMD; Xu et al., 2023). Unlike CNN, which focuses on local receptive fields, Transformer can better capture long-distance dependencies in images through its self-attention mechanism. This has potential advantages in identifying signs that require global context connections, such as diffuse, indistinct millet-like shadows or mediastinal lymph nodes enlargement in pediatric pulmonary tuberculosis. However, ViT has a greater demand for data volume, which is a significant challenge in the field of pediatrics. Secondly, image segmentation is an important task in computer vision. It can divide an image into several parts, each representing a different object or region. In medical image analysis, image segmentation can be used for detecting lesions, diagnosing diseases, and other aspects. In natural image processing, image segmentation is often used in fields such as object recognition and scene understanding. However, due to the significant data differences between medical and natural images, traditional image segmentation methods often require a large amount of labeled data and complex network structures, making it difficult to be widely applied in practical applications. With the development of deep learning technology, an increasing number of researchers have begun to explore how to utilize pre-trained models to enhance the effectiveness and efficiency of image segmentation. Among them, self-supervised learning is a common method, which can learn effective image feature representations in an unsupervised way without the need for a large amount of labeled data (Carol Praveen et al., 2025).

3D printing is redefining the landscape of pediatric tuberculosis treatment by enabling personalized drug delivery, improving surgical plans and tailoring educational tools. Using 3D printing technology to manufacture drug implants or inhalation carriers with complex microstructures (such as porous and sustained-release). In the future, it may be possible to directly print anti-tuberculosis drugs into inhalation formulations suitable for specific airway sizes in children, or implant them into the lesion site for long-term targeted release, thereby increasing local drug concentration, reducing systemic side effects and treatment cycles. In the field of diagnostics, a team developed an inverted microscope using 3D-printed components to support the determination of MODS for tuberculosis culture analysis. Another project combines portable ultraviolet fluorescence devices with 3D-printed shells and smartphone-assisted imaging for sunrise SmartAmp nucleic acid testing. Although 3D printing technology shows promise, only a small portion has reached mass production or regulatory review. Most devices require specialized equipment and formula expertise, and the variability of printing conditions can affect repeatability. Before the wide deployment of these solutions, quality control and biocompatibility must be verified, especially in pediatric settings where dose accuracy and safety are of critical importance (Singh et al., 2025).

Finally, multimodal learning technology based on deep learning can effectively integrate the potential association patterns among heterogeneous data sources, thereby achieving more accurate and personalized disease diagnosis and risk prediction. A study has proposed a new General radiology basic Model (RadFM), which integrates large-scale 2D and 3D medical imaging data to address the long-standing problem of multimodal data shortage in the field of medical imaging AI (Wu et al., 2025). The RadFM Model consists of three main modules: the Visual Encoder, the Perceiver Module and the Large Language Model (LLM). Among them, multimodal fusion aims to achieve the natural fusion of visual and text information in LLM through self-attention mechanisms. Visual embedding and text embedding are interwoven in the sequence, enabling LLM to consider the information of both modalities simultaneously to generate responses. By using large-scale MedMD datasets during the pre-training stage, the model accumulates knowledge of medical-specific terms and images. The domain-specific instruction fine-tuning phase employs the carefully selected radiology student set RadMD to enhance the model's performance in real-world clinical scenarios through high-quality language instructions and responses.

RadFM can not only handle various types of medical images (such as X-ray, CT, MRI, etc.), but also support multi-image input and text interleaving, significantly enhancing the flexibility and applicability of medical image AI. The RadFM model, by integrating medical image and text information, can generate detailed diagnostic reports and inferential diagnoses, providing strong decision support for doctors. Through systematic experiments and evaluations, the accuracy rates (ACC) of RadFM on five diagnostic datasets were 59.96%, 68.82%, 56.32%, 83.62%, and 72.95%, respectively, significantly higher than those of other basic models. On the PMC-VQA dataset, the BLEU score of RadFM was 17.99. However, the scores of other models are mostly below 10. On the MIMIC-CXR dataset, RadFM's BLEU score was 10.21, significantly higher than that of other models. It comprehensively demonstrated the outstanding performance of the RadFM model in the field of radiology. Whether in terms of performance on public datasets, comparison with existing base models, or evaluation on the RadBench benchmark, RadFM has demonstrated significant advantages. Meanwhile, task-specific fine-tuning and human evaluation results further verified the effectiveness and practicality of RadFM. These research results provide strong support for the development and application of general basic models in radiology, as well as for the clinical application of medical AI technology.

However, the application of these AI technology models in pediatric pulmonary tuberculosis still requires further verification and exploration. So far, there have been relatively few studies and literature reports on deep learning AI specifically for pediatric pulmonary tuberculosis. This highlights the lack of clinical and scientific research on AI in the specific field of pediatric imaging.

### 4.2 Laboratory diagnosis

Artificial intelligence-enabled clinical decision support systems have enhanced the influence of laboratory diagnostics in disease risk prediction and clinical decision-making, particularly in areas such as acute kidney injury, septicemia, liver fibrosis, kidney stone, and cardiovascular disease. However, there have been few reports on their application in the auxiliary diagnosis of tuberculosis (Cao and Yi, 2025; Ghanem et al., 2024). In the field of tuberculosis, AI is primarily used for cytokines detection and drug resistance prediction. A study collected data on the levels of 47 serum cytokines in peripheral blood from patients with active tuberculosis and inactive tuberculosis, combined with stimulation of peripheral blood using the tuberculosis antigen ESAT6/CFP10 fusion protein, and analyzed these cytokines data using a machine learning model. This approach can assist in distinguishing between active tuberculosis and latent infection, with a diagnostic accuracy rate exceeding 88% (Xiao et al., 2024).

Genetic gene can also serve as a diagnostic tool for tuberculosis, and an increasing number of studies are utilizing AI to predict *Mycobacterium tuberculosis* (MTB) drug resistance based on bacterial genome data. MTB undergoes a small number of genetic mutations during reproduction and division, leading to resistance to certain anti-tuberculosis drugs. These mutations can also result in different changes during strain propagation. Rapid molecular detection based on genome information is faster and more effective than detection based on culture, and has been widely applied in detecting tuberculosis drug resistance (Cohen et al., 2019). Therefore, some AI systems based on gene sequences have been explored to identify drug resistance in MTB.

Yang et al. (2019) developed an end-to-end deep learning model called DeepAMR, which classifies MTB drug resistance in the presence of coexisting drug resistance by inputting genome data from tuberculosis patients. The results showed that this model outperformed other models in predicting drug resistance rates for four first-line drugs, multiple drug- resistant tuberculosis (MDR-TB), and pan-susceptible tuberculosis, with an average AUROC ranging from 94.4% to 98.7%. It also achieved the highest sensitivity for all drugs except rifampicin (RIF), with values of 94.3%, 91.5%, 87.3%, and 96.3%, respectively. Additionally, the team represented MTB's genetic data as a graph and employed a deep graph learning the heterogeneous graph attention network (HGAT-AMR) model, to predict drug resistance against tuberculosis (Yang et al., 2021). The results showed that the model performed best in tests with isoniazide and rifampicin, with AUROC values of 98.53% and 99.10%, respectively, and achieved the highest sensitivity for the three first-line drugs (isoniazide 94.91%, ethambutol 96.60%, and pyrazinamide 90.63%), and successfully identified genes and single-nucleotide polymorphisms associated with drug resistance. Deelder et al. (2019) combined machine learning models with whole genome sequence (WGS) methods to analyze 16,688 MTB isolated strains to predict drug resistance and detect all mutations associated with drug resistance, aiming to leverage the added value of machine learning. The results showed the highest predictive performance for first-line drugs amikacin, kanamycin, ciprofloxacin, moxifloxacin, and multidrug-resistant tuberculosis (area under the ROC curve exceeding 96%).

Thus, it can be seen that combining AI with laboratory diagnostic results for the auxiliary diagnosis of tuberculosis is an important direction in the development of medical technology, especially of great significance for regions with limited resources and complex cases. In the future, in-depth research can be conducted in areas such as AI automatic image recognition and analysis with microscopic examination of sputum culture, AI clinical microbiological identification systems with automatic recognition of MTB culture dishes (Brenton et al., 2020).

### 4.3 Clinical data

Clinical data can also assist in the diagnosis of tuberculosis to a certain extent. In 2023, the estimated number of new tuberculosis cases worldwide among HIV-infected individuals was approximately 650,000, accounting for approximately 6.0% of the total number of new tuberculosis cases globally (World Health Organization, 2025). Tuberculosis is extremely common among HIV-infected individuals/AIDS patients, being the most common opportunistic infection and as a leading cause of death among AIDS patients. Due to its non-specific early symptoms and delayed diagnosis and treatment, the mortality rate among AIDS patients with tuberculosis is significantly increased. Currently, tuberculosis remains the primary cause of hospitalization and death among HIV-infected patients (both adults and children) worldwide (Ford et al., 2016).

Rajpurkar et al. (2020) utilized chest X-rays (CXRS) and certain clinical data (including age, body temperature, hemoglobin, and white blood cell counts, etc. from 677 HIV-positive patients across two hospitals) to develop a deep learning algorithm named CheXaid. The use of this algorithm improved the diagnostic accuracy of clinicians for tuberculosis, outperforming AI-assisted clinicians (accuracy of 0.79/0.65, *P* < 0.001). Additionally, the training strategy of incorporating clinical variables with CXR enhanced the algorithm's performance in this study, highlighting the importance of integrating inputs in various ways to enhance model efficacy. Gerard et al. (2024) proposed an ontology-based smart auxiliary diagnosis system (SAI) for tuberculosis diagnosis by collecting all auxiliary clinical examination data from patients and analyzing them in conjunction with biological data. The results showed that the system performed well. In the future, the system will combine clinical reports and chest X-rays from the same patient list and utilize time series methods for prediction to address the issues of data complexity and inaccuracy.

Currently, there are few research reports on the use of AI in the auxiliary diagnosis of tuberculosis that extract relevant high-risk behaviors and basic examination results from clinical texts to identify high-risk patients. It may still be necessary to strengthen theoretical research on deep learning models, combine complementary multimodal medical image datasets and image structures, improve the feature extraction capabilities of deep learning models, and thereby improve the accuracy of feature extraction classification.

### 4.4 Pathological examination

Pathological diagnosis is also one of the important means for clinically diagnosing tuberculosis. Currently, the diagnosis of tuberculosis pathology primarily relies on traditional pathological examination as the core method, involving direct observation of morphological changes in pathological tissues. This is also the primary method for distinguishing tuberculosis from other diseases (Yu, 2023). The effective integration of pathological diagnosis and minimally invasive biopsy technique has further reduced the occurrence rate of misdiagnosis and missed diagnosis. However, obtaining pathological samples involves invasive procedures, which significantly limit the widespread adoption of this method (Huang et al., 2023). Currently, AI-assisted pathological diagnosis has also become a mainstream trend. Xiong et al. (2018) developed a CNN model (TB-AI) using a training set of 45 cases (30 positive) and a test set of 201 cases (108 positive). When comparing TB-AI's diagnostic results with those confirmed by pathologists using both microscopes and digital slides, TB-AI achieved a sensitivity of 97.94% and a specificity of 83.65%, but it still has the limitation of insufficient experimental data. Zaizen et al. (2022) and Du et al. (2024) explored the application of AI-assisted pathology in transbronchial lung biopsy (TBLB). The results showed that when detecting acid-fast bacilli (AFB) alone, the sensitivity of AI-assisted examination was significantly higher than that of conventional bacteriological examination (86% vs. 29%, *P* = 0.046), but the specificity of AI-assisted pathology was 100%.

In summary, AI-assisted pathology examination is more sensitive than traditional bacteriological examination in identifying acid-fast bacilli in samples collected by bronchoscopy, and the use of AI-powered microscopy can enhance the sensitivity of sputum smears. This demonstrates that digital pathology is also evolving. Although there are many challenges in this field at present, AI-based methods hold significant potential for application in digital pathology (Shafi and Parwani, 2023; Table 1).

**Table 1:** The application of artificial intelligence in the auxiliary diagnosis of tuberculosis.

**Application fields**	**Author**	**Modal/system**	**Purpose**	**Result**
Medical imaging	Megan Palmer (2023)	CAD4TB	Measure and optimize the performance of an adult CAD system, CAD4TB, to identify tuberculosis on chest x-rays from children with presumptive tuberculosis	Demonstrated a significant improvement in the performance of CAD4TB after fine-tuning with a set of well-characterized pediatric chest x-rays (AUC to 0.72) CAD has the potential to be a useful additional diagnostic tool for pediatric tuberculosis (Palmer et al., 2023)
	Mayidili Nijiati (2022)	ResNet VGG AlexNet	Three different DCNN algorithms, including ResNet, VGG, and AlexNet, were trained to classify the chest radiographs as images of pulmonary TB or without TB	The ResNet algorithm-based AI diagnosis system provided accurate TB diagnosis, which could have broad prospects in clinical application for TB diagnosis, especially in poor regions with high TB incidence (Nijiati et al., 2022)
	Li Wang (2021)	3D-ResNet	To develop and evaluate the effectiveness of a deep learning framework (3D-ResNet) based on CT images to distinguish non-tuberculous mycobacterium lung disease (NTM-LD) from Mycobacterium tuberculosis lung disease (MTB-LD)	The AUCs of 3D-ResNet on training, validating, and testing datasets were 0.90, 0.88, and 0.86, respectively, while the AUC on the external test set was 0.78. The efficacy of 3D-ResNet as a rapid auxiliary diagnostic tool for NTB-LD and MTB-LD. Its use can help provide timely and accurate treatment strategies to patients with these diseases (Wang et al., 2021)
	Zhizhen Qin (2021)	CAD4TB InferRead DR Lunit INSIGHT CXR JF CXR-1 qXR	Evaluate five commercial AI algorithms for triaging tuberculosis using a large dataset that had not previously been used to train any AI algorithms	Only qXR and CAD4TB met the TPP at 90% sensitivity. All five AI algorithms reduced the number of Xpert tests required by 50% while maintaining a sensitivity above 90%. All AI algorithms performed worse among older age groups (>60 years) and people with a history of tuberculosis (Qin et al., 2021)
	Luyao Ma (2020)	U-Net	Develop an automated detection system based on AI in this study to simplify the diagnostic process of active tuberculosis (ATB) and improve the diagnostic accuracy using CT images	For an independent test, the AI system yields an AUC value of 0.980. the AI system performs well for detection of ATB and differential diagnosis of non-ATB (Ma et al., 2020)
Laboratory examination	Xiao JD (2024)	MPGA IMPGA PSO PCC	Conduct a cytokine assay in conjunction with machine learning algorithms to analyze cytokine levels in suspected tuberculosis patients, thereby facilitating the auxiliary diagnosis of active tuberculosis	Analyzing the selection results of the IMPGA-SVM method, it becomes apparent that the frequency of monokine induced by γ-interferon T (MIG-T) markedly surpasses that of other features. This underscores the pivotal role played by MIG in the prediction of active tuberculosis in patients (Xiao et al., 2024)
	Yang Y (2019)	DeepAMR	Propose a multi-task model with deep denoising auto-encoder (DeepAMR) to predict resistance of four first-line anti-TB drugs simultaneously and develop DeepAMR_AMR, a clustering variant based on the DeepAMR, to learn clusters in latent space of the data	The comparison results showed that DeepAMR outperformed the other models with best AUROC for all four first-line drugs, as well as MDR-TB and PANS-TB. It also achieved the highest sensitivity for all drugs except RIF (Yang et al., 2019)
Clinical data	Napthaline GERARD (2024)	SAI	Create Symbolic Artificial Intelligence system (SAI) to diagnose PTB using clinical and paraclinical data	This system contains eight categories of paraclinical tests, containing each different test type. By evaluating our PTB ontology, most patients who did not get TB positive by the microbiological test, have negative results for other types of tests (Gerard et al., 2024)
	Pranav Rajpurkar (2020)	CheXaid	Developed a deep learning algorithm to diagnose TB using clinical information and chest x-ray images from 677 HIV-positive patients with suspected TB from two hospitals in South Africa	Deep learning assistance may improve clinician accuracy in TB diagnosis using chest x-rays, which would be valuable in settings with a high burden of HIV/TB co-infection. Moreover, the high accuracy of the stand-alone algorithm suggests a potential value particularly in settings with a scarcity of radiological expertise (Rajpurkar et al., 2020)
Pathological examination	Yan Xiong (2018)	TB-AI	Built a convolutional neural networks model, named TB-AI, specifically to recognize MTB. Compared the diagnosis of TB-AI to the ground truth result provided by human pathologists, analyzed inconsistencies between AI and human	Examined against the double confirmed diagnosis by pathologists both via microscopes and digital slides, TB-AI achieved 97.94% sensitivity and 83.65% specificity. TB-AI can be a promising support system to detect stained TB bacilli and help make clinical decisions. It holds the potential to relieve the heavy workload of pathologists and decrease chances of missed diagnosis (Xiong et al., 2018)
	Jingli Du (2022)	HALO AI	Forty-two patients underwent bronchoscopy, and were evaluated using AI-supported pathology to detect AFB. The AI-supported pathology diagnosis was compared with bacteriology diagnosis from bronchial lavage fluid and the final definitive diagnosis of mycobacteriosis	The sensitivity of bacteriology and AI-supported pathology was 29% and 86%, respectively. The specificity of AI-supported pathology was 100% in this study. AI-supported pathology may be more sensitive than bacteriological tests for detecting AFB in samples collected via bronchoscopy (Du et al., 2024)

## 5 The prospects and challenges of AI in the auxiliary diagnosis of pediatric pulmonary tuberculosis

### 5.1 Limitations

When using AI for the auxiliary diagnosis of pulmonary tuberculosis, especially for children patients, there are many similar challenges faced with the application of AI in other diseases, such as the immature development of some AI technologies themselves, the need for innovation in application scenarios, data heterogeneity and data island, the lack of external verification, overfitting, domain shift, and the influence of ethical issues in intelligent healthcare (Dodd et al., 2017; Zhao and Cai, 2024; Liu et al., 2022).

#### 5.1.1 Weak data foundations

Currently, there are relatively few AI training datasets available for the diagnosis of pediatric pulmonary tuberculosis, and the types of data are limited, which restricts the performance and generalization ability of AI models. This is mainly reflected in the following aspects: Small sample sets are difficult to cover all the characteristics and heterogeneity of the children's population, which limits the clinical generalizability and generalization ability of the model, increases the transparency and credibility of the imaging model, and raises the uncertainty of prediction. Secondly, the small sample set limits the model's ability to distinguish real signals from noise, leading to frequent overfitting or underfitting phenomena, resulting in a decline in discriminative ability and an increased risk of misjudgment. The essence of domain shift is the distribution difference between the source domain and the target domain. The small sample set makes it difficult for the model to accurately estimate this distribution difference, resulting in poor domain alignment (Fonseca et al., 2020). AI models trained on adult data have a sensitivity of only 36% in children, highlighting the importance of transferability. This inherent data deficiency severely constrains the initial training and iterative optimization of models (Chng et al., 2025).

In addition, there is a lack of external validation of AI models. This type of model, which lacks external validation due to the small number of validation studies, single data sources, insufficient sample sizes, and incomplete evaluation indicators, cannot be widely applied in actual medical scenarios, resulting in a lag in the transformation of AI technology in areas such as disease diagnosis and treatment decision-making. It is impossible to accurately assess the performance fluctuations of the model in different environments, which may lead to problems such as misdiagnosis and missed diagnosis in practical applications, increasing medical risks and affecting the long-term development and social acceptance of AI technology (Kim et al., 2021).

The training datasets for diagnosing pulmonary tuberculosis in children should include multiple centers, use equipment from multiple suppliers and different models from various manufacturers to ensure its accuracy and reliability. Multi-center collaboration is to enhance the promotion of AI models to a wider range of scenarios and fields. Different centers can adapt and optimize the model according to their own needs to accelerate the application of the technology in actual business. There are differences in data sources, collection environments and sample characteristics among different centers. Multi-center collaboration can enhance data diversity and representativeness, enabling models to better adapt to the complexity of the real world. Multi-center collaboration forces models to be trained and validated on diverse data, thereby enhancing their performance in unknown data or new environments, promoting knowledge sharing and collaborative innovation, and reducing the risk of overfitting. Multi-center validation is an important step in evaluating model performance. By conducting tests on data from multiple independent centers, it helps to establish the credibility and reliability of the model. However, multi-center collaboration still faces numerous difficulties at the data and model training levels, including data heterogeneity, data privacy and security, data quality and integrity, model generalization ability, model consistency and interpretability, as well as at the organizational and management levels and technical talent levels in the development and application of AI models (Wan and Shen, 2022; Zhang et al., 2024).

#### 5.1.2 Insufficient technical adaptability

The most common type of pediatric tuberculosis is primary pulmonary tuberculosis, with invasive lesions in the pulmonary parenchyma, mediastinal lymph nodes, and enlarged hilar lymph nodes being the most common imaging manifestations. However, confirming hilar lymph node enlargement on chest X-rays requires diagnostic experience, as lymph node enlargement in children may be obscured by blood vessels, other mediastinal structures, and the thymus. Secondly, a challenging issue in pediatric CT examinations is the control of motion artifacts. Factors such as crying and uncooperative breathing in children are important causes of motion artifacts, and how to suppress or reduce these artifacts during scanning is an issue that needs to be considered. The most critical point is the lack of integration and utilization of multimodal data. Tuberculosis diagnosis requires the integration of imaging, laboratory tests, and electronic health records for diagnosis, but existing AI models lack cross-modal reasoning capabilities. Latt et al. (2024) applied AI models to HIV-positive children, but due to immune suppression altering lesion characteristics, the model's recognition accuracy dropped by 28%. This technical rigidity makes it difficult for algorithms to address the physiological peculiarities of the children.

#### 5.1.3 Opportunities and challenges of federated learning

With the continuous integration of the Internet of Things and AI, Federated Learning (FL), as a cutting-edge machine learning paradigm, enables collaborative training among decentralized devices. FL enables users to jointly build AI models without sharing local raw data, thereby ensuring data privacy, network scalability, and minimizing data transmission costs. The sharing of medical data mainly stems from the fact that high-quality AI models require a large amount of diverse data for training. The protection of patients' privacy is an absolute red line in data security laws. The goal of FL is to achieve collaborative modeling of multi-party data under the premise of ensuring absolute data security. An initial model is distributed to various data sources, and after local training, only the updates of the model are encrypted and uploaded to the central server for aggregation to generate a more powerful global model. FL, as a key branch of privacy-preserving machine learning, can be applied to medical image analysis, genomics and rare disease research, cross-institutional clinical prediction models, pharmaceutical development and real-world research, etc. It provides a practical and feasible technical path for resolving the contradiction between “data silos” and “privacy security” in the medical field. However, in actual work, due to privacy regulations, data security and competitive relationships, hospitals and research institutions have formed insurmountable “data silos,” making it difficult to share and use data. In addition, the distributed nature of the federated learning framework makes it more difficult to deploy defense measures. Issues such as communication efficiency, data and system heterogeneity, and whether user privacy and algorithm robustness can be ensured while using low computing costs, as well as whether decentralized FL can defend against attacks, will be new directions in the field of FL (Arbaoui et al., 2025).

#### 5.1.4 Model interpretability

Although the current mainstream large language models have excellent performance, their internal working mechanisms are like a “black box,” making it difficult to track and understand the decision-making logic. This opacity poses serious risks in high-risk fields such as healthcare, hindering the wide application of AI technology. When using neural networks, we can evaluate the performance of the model through its accuracy. However, when it comes to computer vision problems, it is not only necessary to have the best accuracy, but also to have interpretability and an understanding of which features/data points are helpful for making decisions. It is more important for a model to focus on the correct features than its accuracy. The main methods for understanding CNN include Class Activation Maps (CAM) and Gradient Weighted Class Activation maps (Gradient Weighted Class Activation Mapping, Grad-CAM) and the optimized Grad-CAM (Grad-CAM++); Circuit Sparsity adopts the native sparsity design concept. By artificially constraining the internal connections of the model, it fundamentally reconstructs the network architecture to achieve true interpretability. SHapley Additive exPlanations (SHAP) is a model interpretation method used to explain the prediction results of machine learning models. Its core idea originates from the Shapley value in cooperative game theory. By decomposes the prediction results into the influence of each feature, it provides global and local interpretability for the model. It includes global feature importance, feature effects and individual predictions. Through SHAP, doctors can see which symptoms or risk factors prompted the AI to make decisions, and patients can obtain more transparent and easily understandable results. However, although the field of AI research has made positive progress in the interpretability of large models, there are still technical challenges in thoroughly understanding the intrinsic operating mechanisms of AI systems. The main issues that need to be addressed include the multiple semantics and superposition phenomena of neurons, the universality of explanatory laws, and the cognitive limitations of human understanding (Yisimitila et al., 2025).

#### 5.1.5 The limitations of clinical generalization ability and applicable scenes

According to the current situation, AI models for the auxiliary diagnosis of primary pulmonary tuberculosis have made some progress in the field of imaging diagnosis. However, further validation and optimization are needed through multi-center, large-scale studies targeting children, particularly in resource-limited, high-burden regions. AI models may also be subject to bias, as insufficiently representative training data may amplify existing biases, leading to algorithmic discrimination that impacts the accuracy of medical decisions and exacerbates health equity issues. This can result in diagnostic errors when treating pediatric patients (Cho, 2021). If unreliable reference standards are used during training, the accuracy of the models constructed by such algorithms cannot be guaranteed in real-world applications.

In addition, low-income regions do indeed face a higher burden of pediatric tuberculosis, which is closely related to factors such as a lack of local medical resources, insufficient diagnostic capabilities, and poor access to treatment. The more fundamental contradiction lies in epidemiological misalignment—pediatric tuberculosis deaths mainly occur in low-income regions, but mainstream AI models are trained based on developed countries and have low recognition rates for characteristics of high-burden regions (such as co-infection with HIV and malnutrition; Fonseca et al., 2020; Ngiam and Khor, 2019).

#### 5.1.6 Ethical issues and security risks

The legal boundaries of responsibility among algorithm developers, hospitals, treating physicians, and patients remain unclear. Medication safety risks are particularly severe, as tuberculosis drugs pose significantly higher toxic side effects for children than adults—the incidence of liver toxicity from rifampicin is three times higher in children than in adults, and streptomycin may cause irreversible hearing loss. AI misdiagnoses could directly lead to overtreatment (Liu and Wan, 2020; Contopoulos-Ioannidis et al., 2004). Data privacy threats are also significant. Current AI ethical frameworks primarily focus on adult health, and there is a lack of systematic research and specific guidelines regarding the risks, privacy protection, and fairness associated with AI technology application for children. Ensuring the privacy and security of child patient information during AI-based data analysis presents a major challenge. Among them, in the development of AI models, it is crucial to use low-dose CT images for training and testing. This requires the algorithm to have stronger denoising and robustness, and it has also sparked ethical discussions on whether models trained with historical standard dose data can be directly applied to the fairness and effectiveness of low-dose new data.

Ethical considerations for pediatric imaging mainly involve the following aspects: The necessity of imaging examinations must be strictly evaluated to ensure that the benefits of the examinations outweigh the potential risks. Radiation-free or low-radiation alternative methods should be given priority to avoid unnecessary radiation exposure to children due to habit or excessive medical treatment. The risks of sedative drugs and the necessity of the examinations also need to be weighed. Secondly, children are more sensitive to radiation and strict protective measures must be taken. They also need to obtain written informed consent from their guardians and ensure that the guardians fully understand the necessity and risks of the examination. During the inspection process, irrelevant personnel should be kept away from children to protect their dignity and privacy. Some imaging tests (such as CT) may increase the risk of cancer in children in the future. Medical institutions should establish a long-term follow-up mechanism, pay attention to the health conditions of children, and provide subsequent guidance and intervention when necessary. In conclusion, children's imaging examinations need to strike a balance between medical needs and ethical responsibilities, always starting from the best interests of children, and ensuring the safety, necessity and rationality of the examinations.

### 5.2 Prospects for AI-enabled auxiliary diagnosis of pediatric pulmonary tuberculosis

Although research and applications of AI in the auxiliary diagnosis of pediatric pulmonary tuberculosis are insufficient, its future development remains promising, primarily in the following three areas, given its strong performance in adult tuberculosis-related research.

First, establish a larger-scale, diverse, and high-quality clinical information data set on pediatric pulmonary tuberculosis, including patients of different ages, genders, races, and geographical backgrounds, as well as multi-source clinical information such as medical history, etiology and imaging. This will enable the transition from single-modality analysis to multi-source data fusion diagnosis, thereby improving the accuracy and generalization ability of the model. Liu et al. (2025) constructed a specialized disease database based on real-world data such as electronic medical records and hospital admission records of pediatric tuberculosis cases, providing a data foundation for studies on clinical characteristics, the effectiveness and efficiency of diagnostic strategies, prognosis, and prognostic factors. Yao et al. (2024) included chest CT images from 133 patients, delineated cavities as target lesions, and extracted radiomics features. They established a clinical model based on age and γ-interferon release assay results, selected 10 radiomics features, and combined them with the clinical model to construct a conjunctive model. In the test set, the diagnostic performance of the radiomic model was higher than that of the clinical model, while the conjunctive model demonstrated the best diagnostic performance, with an AUC, sensitivity, specificity, and accuracy of 99.50%, 94.12%, 100.00%, and 96.77%, respectively. Further, it is suggested that future efforts to integrate imaging with clinical and laboratory information to construct combined diagnostic models may offer a breakthrough method to overcome the limitations of imaging-only diagnosis.

Simultaneously, by leveraging Transformer deep learning models to perform in-depth analysis of electronic health records (EMR), key clinical data such as tuberculosis exposure history can be extracted, potentially expanding diagnostic criteria from single imaging findings to a multi-dimensional evidence chain. Sun et al. (2025) proposed a cancer pre-screening method based solely on EHR coding sequences, utilizing autoregressive Transformers for large-scale training and fine-tuning of medical events to enable early prediction of cancer. Additionally, as a cutting-edge direction in AI research, the meta-learning framework enables rapid learning by transferring knowledge from pre-trained models to new tasks. This grants models the ability to generalize quickly in small-sample scenarios, thereby improving diagnostic accuracy on small datasets.

However, generative adversarial networks (GAN) have the potential to accurately reconstruct representative images from the potential pathological, genomics, and imaging features of pediatric pulmonary tuberculosis, solving the problem of the scarcity of extrapulmonary tuberculosis samples in children. Yan et al. (2021) constructed the CycleGAN model using a generative adversarial network, which can generate diagnosable denoised images from ultra-low-dose chest CT images. It achieves superior performance in both subjective and objective evaluations compared to traditional CT iterative denoising algorithms, and can be used for the assessment of pulmonary tuberculosis. Shao et al. (2024) innovatively developed the Multimodal Integration (MMI) model by using multi-dimensional information such as clinical texts, imaging images, and test indicators of 24,107 inpatients, achieving precise prediction of pulmonary infectious diseases and pathogen types, and timely early warning intervention for critical cases. The AUC, accuracy, sensitivity, and specificity of its model in the task of identifying different types of pulmonary infectious diseases were 0.910, 0.848, 0.846, and 0.847, respectively. Moreover, the performance of the MMI model in the diagnosis of pulmonary infectious diseases can be comparable to that of senior clinicians with rich clinical experience. These indicate that the MMI model has potential clinical application value in the auxiliary diagnosis of pulmonary infectious diseases.

**Figure 2:**
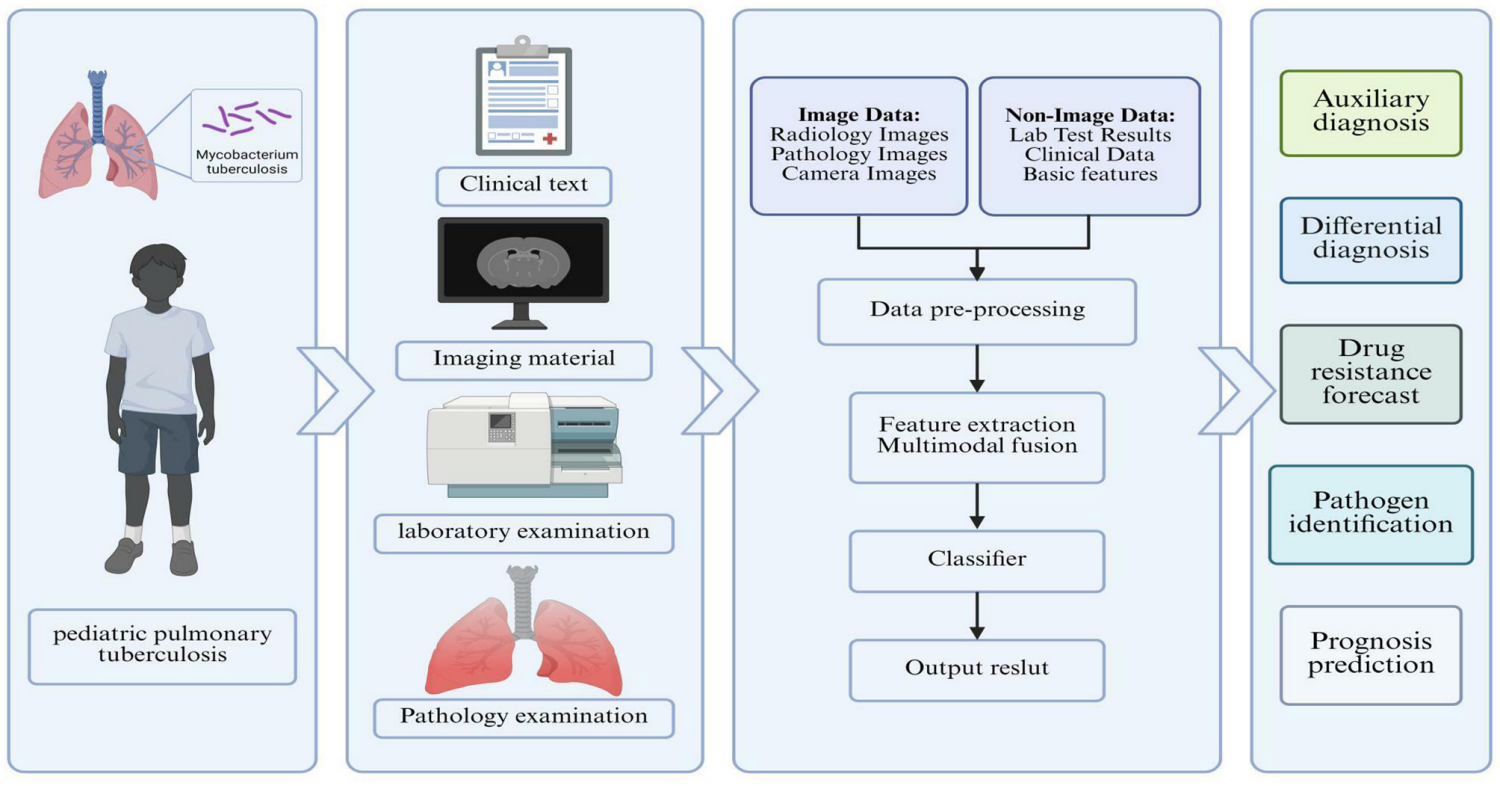
Multimodal integration model for auxiliary diagnosis and treatment of pediatric pulmonary tuberculosis.

It can thus be seen that in the future, AI can be linked to the prognosis assessment, drug resistance prediction, and disease progression information of pediatric pulmonary tuberculosis, and clinical tools suitable for the diagnosis, control, assessment, and follow-up of pediatric pulmonary tuberculosis can be developed (Figure 2).

Second, most current studies are based on single-center data, necessitating further external validation studies. By collaborating with different medical institutions and adopting a multi-center joint research model, the accuracy and reliability of AI models in child patients can be ensured. Especially for pediatric tuberculosis—a disease influenced by three variables: regional epidemiology, equipment conditions, and population characteristics, the forced deployment of models lacking multi-center validation may exacerbate inequalities in the allocation of medical resources. Additionally, attention should be paid to bias issues in AI algorithms. Clinical and radiology physicians should provide more relevant information to help establish low-bias, high-performance AI models.

Third, with the rapid development and widespread application of AI, people are increasingly concerned about the risks associated with AI. Currently, medical AI can be applied to various new scenarios, giving rise to new diagnostic and treatment methods and technologies that enhance human health. However, it still faces numerous challenges. To address the ethical issues related to AI-enabled diagnosis and treatment of pediatric pulmonary tuberculosis, such as the replacement of human intelligence, application pathways, and human-machine relationships, and to foster harmonious doctor-patient relationships, it is essential to strengthen medical ethics training, promote the development of proper ethical perspectives among medical professionals and researchers, and conduct health education campaigns to enhance public awareness and acceptance of medical AI. Based on the WHO's 2021 guideline on “Ethics and governance of artificial intelligence for health” (World Health Organization, 2024; Wei et al., 2022), it is necessary to establish a comprehensive responsibility and penalty mechanism and clear ethical assessment standards for medical AI to strengthen ethical protection for tuberculosis patients and ensure the ethical and safe application of medical AI.

## 6 Conclusion

This article systematically reviews the diagnostic predicaments of AI technology in the auxiliary diagnosis of pediatric pulmonary tuberculosis, as well as its technical applications in medical imaging, laboratory diagnosis, clinical texts and pathology. Although AI methods represented by deep learning have shown great potential in improving the efficiency of image interpretation and mining complex diagnostic patterns, their clinical transformation still faces structural challenges. These challenges are rooted in core deficiencies such as the weak data foundation of pediatric medical, insufficient technical adaptability, lack of interpretability, limited clinical generalization ability and applicable scenarios, as well as ethical and safety risks. Through a critical analysis of existing research, we have found that there is currently a lack of publicly available, large-scale, well-labeled, and multi-center datasets covering different epidemiological backgrounds for the specific disease of pediatric tuberculosis, which leads to a gap in data infrastructure, significantly limited accuracy, reliability, and generalization ability of the model, as well as insufficient multimodal fusion capability. Most research on explainable AI still remains at the level of technical demonstration and lacks application in real clinical scenarios, resulting in a gap in the integration of technology and clinical practice. Existing research is highly focused on the “diagnosis” stage, while the exploration of AI in the full-process management of early screening, therapeutic effect evaluation, prognosis prediction and drug resistance inference of pediatric pulmonary tuberculosis is still very limited.

To address the above challenges, we have discussed the future research routes. Firstly, it is necessary to establish multi-center cooperation that adheres to ethical and legal norms, explore the use of technologies such as federated learning, and build representative high-quality large databases under the premise of protecting privacy, laying the foundation for model development. Secondly, promote “clinical-centered” XAI research, design clinical trials, and quantitatively evaluate the practical effectiveness of tools such as SHAP in reducing the misdiagnosis rate of doctors, shortening the time for reading films, or improving the management decisions of children patients. Explore the application of new architectures such as Visual Transformor and multimodal large models in pediatric pulmonary tuberculosis, correlate clinical information with imaging and laboratory diagnostic results, enhance multimodal fusion capabilities, and construct joint diagnostic models to provide more accurate diagnoses for pediatric pulmonary tuberculosis. Finally, it is necessary to extend the application scenarios of AI as much as possible from diagnosis to the front and back, truly achieving the empowerment of AI for the entire chain of “prevention—diagnosis—treatment—management” of pediatric pulmonary tuberculosis. In conclusion, AI technology is expected to revolutionize the diagnosis and treatment model of pediatric pulmonary tuberculosis. However, its success depends on close cross-disciplinary and cross-institutional cooperation, as well as a fundamental shift from pursuing model performance to solving practical clinical problems and building reliable and trustworthy systems. Future work should be dedicated to filling the aforementioned gaps and, along a clear roadmap, promoting the transformation of AI from a laboratory tool to an accessible, reliable and trustworthy clinical support partner.
